# Proceedings October 3d, 1876

**Published:** 1876-10

**Authors:** 


TUESDAY EVENING, October 3d.

The President, Dr. Wyckoff, in the chair.

Members present:—Drs. Coxe, O'Brien, Brecht, Bartlett, Brush and Howe. By invitation, Dr. McCartey.

The minutes of last meeting were read and approved.

DR. COXE read a paper on the "Toxic effect of Lead."

DR. BARTLETT said he was much pleased with the practical character of Dr. Coxe's paper. Had seen several cases of lead poisioning, and some in which it could be suspected. Saw some time ago a woman, wife of a workman, in the the Cornell Lead works, who had become poisoned, no doubt, by handling and washing her husbands clothes. He thought that much stress should be laid upon the prophylactic measures to be adopted.

On motion, a vote of thanks was tendered Dr. Coxe for his intеesting paper.

DR. E. R. BARNES was appointed essayist for the next meeting.

DR. HOWE said that he had a method of illumination to present which he thought would be of interest on account of its cheapness and ease of construction. Dr. Howe exhibited its advantage in the illumination of the eye and throat. It consisted in an ordinary dark lantern fitted over the chimney of a student lamp. To focus the light on the cornea so as to be available in removing foreign bodies, etc., at night, a lense was fitted to the head band of a laryngoscopic mirror on a movable arm, thus leaving both arms of the operator free, when this was used on the patient.

DR. O'BRIEN under the head of prevailing diseases spoke of diptheria as prevailing to some extent, dysentery and whooping cough were prevailing.

DR. BARNES said as he came in late he would like to ask a question on the subject of the paper. Said he thought insanity from saturnismus was rare; had never heard of a case from professional sources. Recently saw a young man who had been engaged in painting for *six* months, using a preparation in turpentine. He became insane and was sent to the Providence Asylum and thence to Utica. He had been informed that the authorities, taking the history of the case in consideration, ascribed its cause to lead poisoning, and would like to ask if the author of the paper had seen similar cases.

DR. COXE narrated one case of acute mania of four days duration which he considered due to lead poisoning.

On motion, the society adjourned.

